# Pharmacological Therapy in Inflammatory Bowel Diseases: A Narrative Review of the Past 90 Years

**DOI:** 10.3390/ph16091272

**Published:** 2023-09-08

**Authors:** Marcello Imbrizi, Fernando Magro, Claudio Saddy Rodrigues Coy

**Affiliations:** 1 Department of Surgery, Faculty of Medical Sciences, University of Campinas, Cidade Universitária Zeferino Vaz-Barão Geraldo, Campinas 13083-970, SP, Brazil; 2 Unit of Pharmacology and Therapeutics, Department of Biomedicine, Faculty of Medicine, University of Porto, 4200-450 Porto, Portugal

**Keywords:** Crohn’s disease, ulcerative colitis, 5-ASA, corticosteroid, immunomodulator, biological therapy, JAK, s1P

## Abstract

Inflammatory Bowel Diseases had their first peak in incidence in countries in North America, Europe, and Oceania and are currently experiencing a new acceleration in incidence, especially in Latin America and Asia. Despite technological advances, 90 years after the development of the first molecule for the treatment of IBD, we still do not have drugs that promote disease remission in a generalized way. We carried out a narrative review on therapeutic advances in the treatment of IBD, the mechanisms of action, and the challenges facing the therapeutic goals in the treatment of IBD. Salicylates are still used in the treatment of Ulcerative Colitis. Corticosteroids have an indication restricted to the period of therapeutic induction due to frequent adverse events, while technologies with less systemic action have been developed. Most immunomodulators showed a late onset of action, requiring a differentiated initial strategy to control the disease. New therapeutic perspectives emerged with biological therapy, initially with anti-TNF, followed by anti-integrins and anti-interleukins. Despite the different mechanisms of action, there are similarities between the general rates of effectiveness. These similar results were also evidenced in JAK inhibitors and S1p modulators, the last therapeutic classes approved for the treatment of IBD.

## 1. Introduction

The history of medicine and the emergence of diseases is intertwined with the history of humanity. The development of sanitary techniques, with the subsequent knowledge of antibiotic therapies, led to a partial decline in infectious and contagious diseases, especially in terms of mortality. Parallel to this event, the socio-environmental changes that occurred after the first and second industrial revolutions contributed to the occurrence of immunological and metabolic changes in individuals, and since the 20th century, we have observed an exponential increase in many diseases, among them, immune-mediated disorders [1,2].

Naturally, a continuous and balanced inflammatory process is observed in the digestive tract, a barrier organ. The interaction between the microbiota and the immune response (especially innate) is harmonious and self-limiting [3]. An imbalance that occurs in genetically prone individuals (genome), exposed to a propitious environment (interactome) causes the modification of the intestinal microbiota (microbiome) and an uncontrolled immune response (immunome) leading to the emergence of Ulcerative Colitis (UC) and Crohn’s Disease (CD): Inflammatory Bowel Diseases (IBD) [4,5,6,7,8].

**Figure 1:**
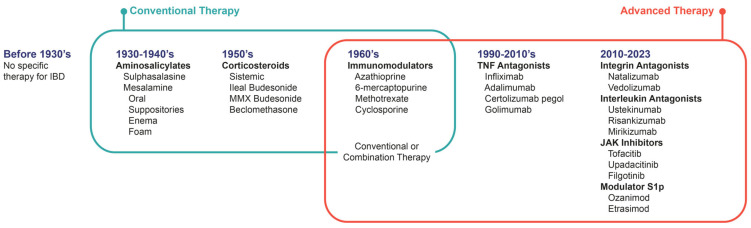
Timeline: development of IBD therapies by decade. IBD: Inflammatory Bowel Disease. MMX: Multi-matrix system. TNF: tumor necrosis factor. JAK: Janus Kinase. S1p: Sphingosine-1-phosphate.

Until the 1930s, no therapy of proven efficacy was used for the treatment of Inflammatory Bowel Diseases. In the first half of the twentieth century, knowledge of these diseases was as scarce as therapeutic options (Figure 1). The first step towards the attempt to control IBD with medication was taken by Nanna Svartz with the development of Sulfasalazine and until the 90′s, other therapeutic classes were developed: glucocorticoids and immunomodulators. This set of therapeutic modalities is currently called “conventional therapies”. From the 1990s onwards, the development of monoclonal antibodies, which were already used in other diseases, (e.g., rheumatologic, and oncologic), showed efficacy in IBD. The first approved monoclonal antibodies were TNF-alpha antagonists (anti-TNF), followed by integrin antagonists (anti-integrins) and more recently interleukin antagonists (anti-IL). The use of monoclonal antibodies in IBD are called “biological therapies”. In the 2010s, there was the development of new synthetic technologies, called “small molecules”, where efficacy was observed in Janus Kinase inhibitors (anti-JAK) and sphingosine-1-phosphate modulators (S1p modulators) [4,6,9].

In the field of IBD in pediatrics, the scenario changes due to the longer time to include this population in clinical trials and, consequently, its on-label indication. In advanced therapies, although only some anti-TNF are formally indicated, other therapies, such as integrin antagonists or anti-interleukin, are routinely used pending the completion of pivotal studies in this population [10,11,12]. Another differential concerns the greater knowledge of dietary therapies such as the Exclusive Enteral Diet and the CD Exclusion Diet [10,13,14].

Considering almost a century of evolution in drug therapies for IBD, we still observe an insurmountable therapeutic ceiling for the drugs approved so far, and we note some discrepancies between the therapeutic objectives in clinical trials and in clinical practice [15,16,17,18]. Based on this statement, the main objective of this review is to approach in a narrative way the mechanisms of action of each therapeutic class and their results when tested in controlled studies. At the same time, we critically evaluated the changes in the main outcomes in clinical trials and the lack of data consistent with clinical practice. Thus, we intend to point out to IBD professionals, in an objective and contemporaneous way, the indications and therapeutic differentials proposed for the treatment of Crohn’s Disease and Ulcerative Colitis.

## 2. Methodology

A non-systematic review of the literature was carried out with the objective of exploring, analyzing, and synthesizing the existing knowledge about the different lines of drug treatment for IBD. A bibliographic search was carried out in the databases: PubMed, Scopus and Web of Science using specific keywords: IBD, Crohn’s Disease, Ulcerative Colitis, treatment, and drugs. We use studies of relevance and notorious knowledge, as well as the use of cross-references. Review, cohort, cross-sectional studies, and clinical trials were included. Considering the mandatory nature of clinical trials since the advent of biological therapy, these were the studies selected to address the main outcomes related to biological therapy and small molecules, as well as address methodological advances and their shortcomings.

Once the studies have been selected, we analyze and summarize their results in a narrative and linear way, providing an overview and evolutionary view of therapies, therapeutic results, therapeutic indications, and gaps that still exist in the literature.

## 3. Therapies and Therapeutic Goals

Therapies aimed at the treatment of IBD, as well as the concepts about therapeutic objectives, are relatively recent. Although the first therapies date back to the first half of the 20th century, the first blind and randomized trials to assess therapeutic efficacy date back to the 1990s [19,20,21]. The drugs recommended for the treatment of IBD are shown in Table 1. Therapeutic studies demonstrated that the absence of symptoms was not capable of preventing the natural progression of IBD, thus, studies with an emphasis on clinical outcomes. Among numerous proposals, the proposal of the International Organization for IBD (IOIBD) is considered among the most relevant, which proposes target-directed therapy (Treat to Target—T2T) considering the short-term objectives (such as clinical response), medium-term (such as reduction in inflammatory markers), and long-term (such as mucosal healing) [16,22]. The same institution also proposes continuous care to patients with the proposal of the SPIRIT consensus, which warns about care in the different manifestations of the disease, even after controlling the inflammatory activity [23].

## 4. Amino Salicylates

**Figure 2:**
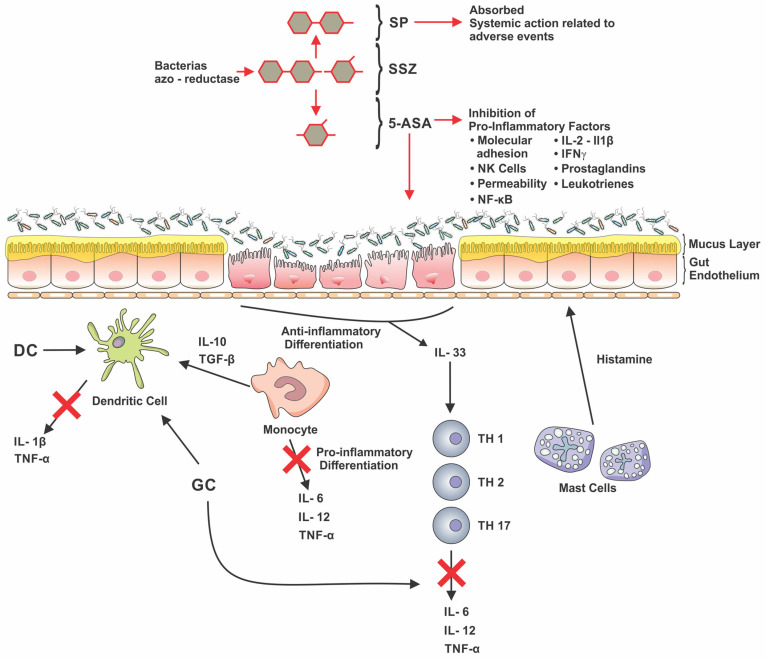
Action of salicylates and glucocorticoids in IBD. 5-ASA acts on pro-inflammatory factors such as molecular adhesion, Natural Killer cell activation, intestinal permeability, nuclear factor kappa B activation, IL-1β, IL-2, Interferon gamma, prostaglandins, and leukotrienes. Sulfasalazine is converted by bacterial azo-reductases into 5-ASA, the UC-acting moiety, and into Sulphapyridine, which is absorbed and is generally associated with adverse events. Glucocorticoids downregulate the inflammatory cascade by reducing cytokines (represented by the red crosses) such as IL-1 beta, IL-6, IL-12, TNF-alpha, in addition to allowing the release of regulatory cytokines such as IL-10. DC: Dendritic cell. GC: glucocorticoids. IL: interleukin. IFN: interferon. *NF-*κB*:* nuclear factor kappa B. NK: Natural Killer cells. SP: sulfapyridine. SSZ: sulfasalazine. TGF-β: transforming growth factor beta. TH: T helper. TNF: tumor necrosis factor. 5-ASA: 5-aminosalicylate.

The first medication directed at the treatment of IBD was sulfasalazine (SSZ), which consists of binding sulphapyridine (active metabolite responsible for most adverse events related to the use of SSZ) and the 5-ASA portion, which is responsible for the anti-inflammatory action [6]. Since then, isolated portions of 5-ASA have been developed, such as mesalamine, olasalazine, and balsalazide [60,61]. The mechanism of action of the medication is partially known and is presented in Figure 2.

The use of salicylates is currently only recommended for the treatment of UC [26,27,28,29]. The meta-analysis performed by Nikfar and colleagues comparing SSZ with 5-ASA showed no differences in efficacy in the treatment of UC [60]. In addition to the effect of salicylates on UC, their action in the prevention of colorectal cancer has been highlighted, although more studies are needed to identify the exact effect on cancer and to identify an ideal model of chemoprevention [62].

Most patients with UC will have mild to moderate manifestations of the disease and tend to respond to 5-ASA treatment. Patients with distal disease may benefit from the use of topical mesalamine therapies alone, while oral therapy is recommended in extensive disease, preferably in combination with the suppository or enema formulations [63]. A systematic review and meta-analysis in the network corroborate these data but point out that there is more evidence that high doses of 5-ASA have more evidence of its effectiveness than combined therapy in left or extensive UC [64]. However, formulations that require a greater number of doses or oral and topical associations reduce therapeutic adherence in approximately 40% of patients. Single daily doses are proven to be better adhered to, even interfering in the reduction in surgeries and hospitalizations [65].

The IMPACT, a phase 4 non-interventional study, evaluated the use of oral prolonged-release mesalamine in patients with mild to moderate UC in a Dutch cohort and found that mesalamine is effective in inducing clinical and endoscopic remission, regardless of the extent of the disease and is associated with low recurrence rates [66].

Most recommendations suggest the use of oral 5-ASA at a dose greater than or equal to 2 g daily, and high doses of mesalamine (greater than or equal to 4g daily) may be used in diseases with greater inflammatory activity and during therapeutic induction. The recommended topical dose (suppository or enema) is usually 1 g daily [26,28].

## 5. Glucocorticoids

Corticosteroids were the second therapeutic class developed that demonstrated efficacy in the treatment of IBD. [67]. This class is effective for therapeutic induction and quick disease control in situations of clinical flares; however, long-term use is not recommended because it is the class with the highest potential for adverse events [28,29].

Glucocorticoids (GC) are endogenous hormones which began to be produced synthetically in the mid-twentieth century. Inflammatory stimuli are one of the mechanisms that stimulate endogenous GC secretion to regulate inflammatory activity. GCs operate in genomic and non-genomic pathways in both the innate and adaptive immune systems. The genomic action acts on genes responsible for pro-inflammatory mediators, such as activator protein-1 and nuclear factor kappa B. Among the non-genomic actions, GC can delay the inflammatory process by generating secondary intracellular transmissions and signal transduction cascades [68]. Figure 2 exemplifies some of the GC action movements.

The first generation of GC is the most used, and the main representatives are prednisone, methylprednisolone, and hydrocortisone [28,29]. There is a consensus among international recommendations that doses equivalent to 1 mg/kg of prednisone, not exceeding 40–60 mg daily, are effective for the treatment of IBD [28,29].

Aiming to reduce the severity of adverse events from glucocorticoids, the second generation of this class was developed to concentrate its action in the gastrointestinal tract and reduce its systemic action [32,69]. Examples of this generation are ileally acting budesonide, budesonide multi-matrix system, and beclomethasone dipropionate. Although the effectiveness of this class does not parallel that of systemic therapy, some phenotypes of CD (e.g., mild to moderately active ileal Crohn’s disease) and UC (e.g., mild to moderate ulcerative colitis unresponsive to 5-ASA) show benefit in the use of this therapy [26,28,70]. The classic study by Campieri et al. compared the action of ileal budesonide with prednisone in ileal CD or ileum and right colon CD, demonstrating similarity in achieving clinical remission with lower rates of adverse events in the budesonide group [71]. The CORE II study randomized, double-blind, double-dummy, placebo-controlled, and parallel-group evaluation of the efficacy of Budesonide MMX using combined clinical and endoscopic remission as the primary endpoint. The results of budesonide MMX and its comparators, the group using ileal budesonide and the placebo group were 17.4%, 12.6%, and 4.5%, the statistical difference being present only in the comparison between budesonide MMX and placebo. Interestingly, the study found that although oral budesonide is a locally acting corticosteroid with poor bioavailability, both ileally acting budesonide and MMX affected baseline cortisol when compared to placebo [31].

## 6. Immunomodulators: Thiopurines and Methotrexate

The first immunomodulatory drugs with the possibility of long-term use, in order to maintain the remission of patients’ refractory to 5-ASA or corticosteroid-dependent or corticosteroid-refractory, were azathioprine, 6-mercaptopurine, and methotrexate, classically named immunomodulators (Figure 1) [6,72]. Immunomodulators have a slow onset of action and should not be used as monotherapy in therapeutic induction [61].

The mechanism of action of thiopurines (azathioprine and 6-mercaptopurine) in IBD is complex and knowledge is incomplete, where several metabolites are involved in several immunosuppressive and cytotoxic mechanisms. Telm et al. mention three more relevant mechanisms: I. The incorporation of 6-thioguanine to DNA or RNA, inhibiting replication, DNA repair, and protein synthesis; II. the metabolite 6-thioinosine 5′-monophosphate causes inhibition of de novo purine synthesis, altering replication; III. apoptosis of T cells via mitochondrial pathway activated by 6-thioguanosine 5′-triphosphate. Several other mechanisms are studied as well as their genetic and metabolic differentials [73].

Thiopurines show efficacy in monotherapy in maintaining remission, they can be used in the prevention of postoperative recurrence, and in association with biological therapies. Studies indicate that the effectiveness of this class is directly related to the levels of 6-thioguanine (6-TGN) [29]. There is less data in the literature on the use of methotrexate in IBD when compared to thiopurines. It is shown to be effective in maintaining remission in Crohn’s disease, and there are few data that suggest its effectiveness in reducing the formation of anti-drug antibodies [74,75].

The OPTIC study was a prospective, randomized, double-blind, placebo-controlled trial that evaluated the efficacy of mercaptopurine in maintaining clinical, endoscopic, and histologic remission following corticosteroid induction. With a total of 59 participants, the 29 patients in the mercaptopurine group had at the end of 52 weeks, rates of 48.3%, 51.7%, and 41.4% of clinical, endoscopic, and histological remission, respectively, while only 10%, 13.3%, and 16.7% of the placebo group achieved the same results [33].

Some considerations about immunomodulators should be remembered. In general, they are safe drugs. However, methotrexate should not be used in pregnant women because it is teratogenic, and the use of thiopurines increases the risk of lymphoma, especially in young men, in both sexes over 50 years old, and individuals that have never been infected by Epstein Barr virus, if they present the first infection [28,29].

## 7. Biological Therapy

**Figure 3:**
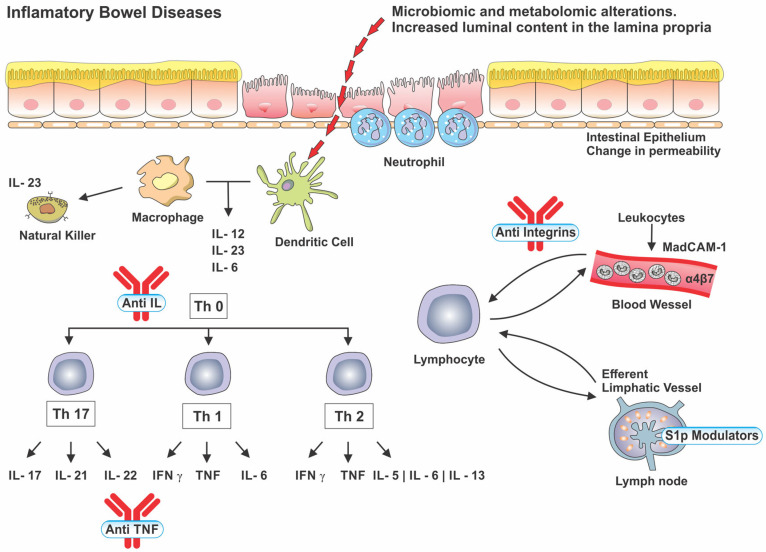
Inflammatory pathways in IBD and areas of action of biological therapies and S1p modulators. IL: Interleukin. Th: T helper. INF: Interferon. TNF: Tumor Necrosis Factor. MadCAM-1: Mucosal vascular addressin cell adhesion molecule 1. S1p: Sphingosine-1-phosphate.

In the 1990s, the advent of immunobiological therapies reached the IBD therapeutic panel. Infliximab ushered in the era of biologics, medications that achieve higher rates of disease remission when compared to conventional therapies and that have reduced the rate of hospitalizations and resections over the past two decades. Technology accompanied the development of these medications, starting from a chimeric molecule to the development of humanized and fully human molecules. Three classes of biological therapies are currently used in the treatment of IBD: (I) anti-TNF, (II) anti-integrins, and (III) anti-interleukins [6,35]. Figure 3 exemplifies an inflammatory process in the intestinal mucosa and the main sites of action of these therapies. We will cite the efficacy results of each therapy preferably in the naive population of biological therapies or small molecules, according to the main outcomes of the pivotal studies.

## 8. Anti-TNF

TNFα is part of a wide network of proteins and receptors involved in immune regulation. This protein is involved in many inflammatory manifestations, such as fever, activation of T cells, and granulocytes. In IBD, this mediator can recruit inflammatory cells, causing edema, angiogenesis, and hypervascularization, activating the coagulation cascade, among others. Specifically in CD, acting in the formation of granuloma [76,77,78].

Infliximab (IFX) is a chimeric mAb (75% human and 25% murine) with high affinity and specificity for TNFα. The ACCENT 1 study demonstrated its efficacy in CD by assessing clinical response at week 2 (achieved by 58% of patients) and clinical remission at week 30 (39%) and loss of response at week 54, which was low in the group with the standard dose of IFX [34]. ACCENT 2 indicated its action in perianal CD with fistulas, where therapeutic response and remission were observed in 46% and 36% of patients [36]. The SONIC trial showed an increase in the effectiveness of IFX when associated with AZA both in clinical remission and in mucosal healing (44% × 56% and 30% × 43% in monotherapy or combined therapy, respectively) [79]. The REACH study showed efficacy and safety of the drug in the pediatric population with CD [80].

In UC, the action of IFX was demonstrated through clinical response and mucosal healing by the ACT 1 and ACT 2 trials. In ACT 1, the clinical response was 69%, 52%, and 45%, and mucosal healing was 62%, 50%, and 45% at weeks 8, 30, and 54. In ACT 2, the clinical response was 64% and 47%, and mucosal healing was 60% and 46% at weeks 8 and 30, respectively [81].

In 2021, a study proved the effectiveness of the subcutaneous formulation of infliximab in the maintenance of therapy in IBD, as well as the safety in switching from the intravenous to the subcutaneous formulation in patients on remission [37,82].

Adalimumab (ADA) is a fully human recombinant immunoglobulin G1 mAb with high affinity and specificity to TNFα. The CLASSIC I and II trials demonstrated the efficacy of ADA in CD reaching 36% clinical remission at week 4 and 79% at the end of 56 weeks (however, with a small number of patients at the end of the study) [38]. The CHARM study evaluated the efficacy of ADA in maintaining response and remission in CD, achieving remission rates of 40% and 36% at weeks 26 and 56, respectively [83].

The action of ADA on UC was evidenced by studies ULTRA 1 and 2. In the ULTRA 1, a clinical response rate to ADA at week 8 of 54.6% was observed with 18.5% of patients in clinical remission [40]. Subsequently, ULTRA 2 showed response and clinical remission at week 52 of 34.6% and 17.3% [40]. The phase 3, double-blind, randomized, multicenter study entitled SERENE UC researched if high doses of Adalimumab in induction and maintenance would increase the UC remission rate. However, high doses did not increase significantly in the therapeutic response rate [84].

Certolizumab pegol is a human monoclonal antibody Fab′ conjugated with polyethylene glycol (PEG), an inert 40-kDa macromolecule. The PRECISE 1 and 2 studies evaluated the effectiveness of Certolizumab pegol in Crohn’s Disease. The studies indicate a clinical response rate of 40% in the bio naive population at week 6. The clinical remission rate at week 6, including patients with previous use of IFX, was 22% [41]. PRECISE-2 evaluated the response rate and clinical remission up to week 26 in a population containing 24% of patients with previous use of IFX. Of responders at week 6, 62% maintained clinical response at week 26. The clinical remission rate at week 26 was 48% in an Intention-to-Treat assessment [42].

Golimumab is a fully human IgG1 kappa monoclonal antibody, which binds to TNF-α bound to the membrane and soluble developed through the technique of producing transgenic human antibodies and has brought several clinical gains, such as greater affinity for TNF-α and lower immunogenicity. The action of Golimumab on UC was demonstrated by PURSUIT SC (induction) and PURSUIT-M (maintenance). Clinical response and remission were seen in 51% and 17.8% of patients at week 6 [43]. Maintenance of clinical response at week 54 occurred in 47% of patients while the clinical remission rate was 23.2% [44].

## 9. Anti-Integrin

Anti-integrins are molecules expressed by leukocytes that bind to adhesion molecules (CAM) and allow the trafficking of these cells [85]. The first drug of this class was Natalizumab (anti-alpha4), intended for the treatment of CD; however, as it lacks specificity for the digestive tract, it was associated with the development of progressive multifocal leukoencephalopathy, and its use is limited or restricted in most countries [86,87]. The ENCORE trial showed a response and clinical remission rate of 60% and 38% on week 8 [45].

Vedolizumab (VEDO) is a humanized mAb capable of specifically blocking the α4β7 heterodimer causing a selective inhibition of intestinal lymphocyte traffic without interfering with traffic to the central nervous system (as occurred with Natalizumab). The α4β7 integrin, located on the leukocyte surface (B and T lymphocytes) specifically interacts with the adhesion molecule CAM-1 (MAdCAM-1) which is predominantly expressed in the vascular endothelium of the digestive tract [88].

The GEMINI 2 and 3 trials showed the effectiveness of Vedolizumab in the treatment of CD. In GEMINI 2, a clinical response and remission rate of 31.4% and 14.5% was observed at week 6. It is important to consider that 67.7% of patients had already used one or more anti-TNF. At the end of 54 weeks, patients using the standard dose of VEDO achieved 43.5% clinical response and 39% clinical remission [46]. GEMINI 3 separates the population into anti-TNF failed and anti-TNF naïve, demonstrating the same clinical response at week 6 (39.2%) but with a higher rate of clinical remission in the anti-TNF naive population (31.4% × 15.2% in those exposed to anti-TNF) [89].

In the GEMINI 1 trial, where the efficacy of VEDO in UC was tested, 47.1% of response and 16.9% of clinical remission were observed at week 6. At the end of 52 weeks, 56.6% and 41.8% of patients were in the response or remission clinic, respectively [47]. The effectiveness of Vedolizumab in UC patients undergoing proctocolectomy with ileal-pouch anastomosis with the subsequent development of pouchitis was evaluated in a phase 4, multicenter, double-blind, randomized, placebo-controlled trial for 34 weeks. The primary outcome was the remission of the disease assessed by the Modified Pouchitis Disease Activity Index (mPDAI). Remission was reached by 31% and 35% of patients using Vedolizumab in weeks 14 and 34, while the placebo group reached 10% and 18%, respectively [90].

Recently, the VISIBLE studies demonstrated the efficacy and safety of the subcutaneous formulation of Vedolizumab in achieving and maintaining clinical remission after induction with intravenous doses [48,91].

## 10. Anti-Interleukin

Interleukins play a prominent role in the pathogenesis of immune-mediated diseases. Specifically, in IBD, interleukins 12 and 23, produced mainly by dendritic cells and macrophages, are involved in lymphocyte differentiation with Th1 and Th17 responses, respectively [92].

Ustekinumab (UST) is a human monoclonal antibody that binds to the p40 subunit of interleukin-12 and interleukin-23. The UNITI-1 trial evaluated the effectiveness of UST in CD patients who had failed previous biological therapy, while the UNITI-2 trial used a bio naive population. Response and clinical remission rates at week 6 were 33.7% and 18.5% in the UNITI-1 and 55.5% and 34.9% in the UNITI-2. At the end of 44 weeks, clinical remission was observed in 41.1% and 62.5% of patients in the UNITI 1 and 2, respectively, who received doses every 8 weeks [49].

UNIFI Induction demonstrated the efficacy of UST in UC, achieving 52% and 62% clinical response and 14% and 24% clinical remission at week 8, in populations exposed to biological therapy and bio naives, respectively. Maintenance of clinical response was observed in 70% and 78%, while clinical remission rates reached 41% and 49% of the population previously exposed or naive to biological therapy at the end of week 44, according to data from the UNIFI Maintenance trial [50].

Risankizumab (RIZA) is an anti-interleukin 23 Mab directed against its p19 subunit. The ADVANCE AND MOTIVATE studies evaluated the efficacy of therapeutic induction of RIZA in CD. The difference between the studies was that in ADVANCE 42% of patients were bio naive while in MOTIVATE all patients had already been treated with some biological therapy. The primary endpoints in the studies were clinical remission and endoscopic response in week 12. In ADVANCE, 47% and 37% of bio-naive or bio-failure patients achieved clinical remission, while 43% and 23% demonstrated endoscopic response at week 12. In MOTIVATE, 40% achieved clinical remission and 34% endoscopic response [51].

The assessment of therapeutic maintenance of RIZA in CD was studied in FORTIFY. In total, 64% of bio-naive and 48% of bio-failures patients experienced clinical remission at week 52, while endoscopic response was achieved in 53% and 44% in the respective groups [52].

Mirikizumab is a p19-driven antibody against interleukin-23. Its efficacy and safety in the treatment of UC were evaluated in the LUCENT-1 (induction) and LUCENT-2 (maintenance) studies. Clinical remission after 12 and 52 weeks using Mirikizumab was 24.2% and 49.9% against 13.3% and 25.1 in the placebo group, respectively [53].

## 11. Small Molecules

The high rate of non-responders to biologic therapy has prompted research into novel signaling pathway blocks, including Janus Kinases (JAK) blockers, DNA transcription activation and transduction signaling (STAT) blockers, and sphingosine-1-phosphate (S1p) modulators. Because they are synthetic molecules, but with greater immunomodulatory potential than conventional therapies, this latest therapeutic generation was called “small molecules” [93].

## 12. JAK Inhibitors

**Figure 4:**
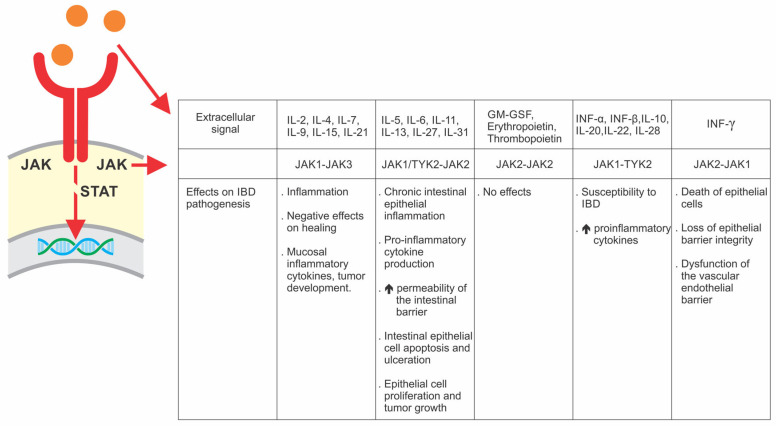
Mechanism of action of JAK inhibitors in IBD [95]. JAK blockade occurs in pairs involving five different inhibition scenarios interrupting STAT-mediated extracellular-nuclear communication. IL: Interleukin; JAK: Janus Kinase; GM-GSF: Granulocyte-macrophage colony-stimulating factor; INF: STAT: Signal transducer and activator of transcription proteins; INF: Interferon. Adapted with permission from Pippis, Elleni J; Yacyshyn, Bruce R, Inflammatory Bowel Diseases; published by Oxford University Press, 2020.

The JAK family is composed of JAK 1, JAK 2, JAK 3, and tyrosine kinase 2 (TKY2). An extracellular signal (cytokine) can bind to these receptors and induce their activation and, consequently, auto-phosphorylation, and/or transphosphorylation with subsequent interaction of the family composed of seven STATs (STAT 1, 2, 3, 4, 5A, and 5B) with subsequent translocation of information to the cell nucleus. JAK signaling occurs in pairs, with different combinations of commands capable of activating and regulating biological processes, such as apoptosis, cell proliferation and differentiation, among others [94]. Figure 4.

Tofacitinib was the first small molecule released for the treatment of UC, it is a molecule that acts on all JAK, mainly on JAK 1 and 3. The OCTAVE 1 and 2 studies evaluated the ability to induce clinical remission at week 8. Previous exposure to anti-TNF occurred in 52% and 53% of groups 1 and 2 and clinical remission was achieved by 18.5% and 16.6% of patients, respectively [54]. Assessment of maintenance of response was assessed by the OCTAVE Sustain trial in which clinical remission at week 52 was the primary endpoint and was achieved by 40.6% of patients not using the standard dose of tofacitinib [54].

Filgotinib is a preferred inhibitor of JAK 1 (blocking potency over JAK 1 five times greater than the others) and has been evaluated in the treatment of UC through the SELECTION trials. Unlike most IBD therapies, there are no differences in induction or maintenance doses. Clinical remission in week 10 was achieved by 26.1% of bio-naive patients and 11.5% of bio-experienced patients. At the end of 58 weeks, 37.2% of patients (both groups) achieved clinical remission [55].

Upadacitinib (UPA) is a selective JAK-1 inhibitor molecule that had its action on the UC evaluated by the U-ACHIVE and U-ACCOMPLISH trials, and the main outcome was clinical remission. Induction of clinical remission was assessed by UC 1 and 2, where 53% and 50% of the population was previously exposed to biological therapy. Clinical remission at week 8 was 26% and 33% in the respective trials. In the maintenance study, 42% of patients using UPA 15mg daily and 52% of patients using UPA 30mg daily achieved clinical remission at the end of 52 weeks [56]. More recently UPA received its regulatory approval for the treatment of CD based on the results of the U-EXCEL and U-EXCEED studies for therapeutic induction, with clinical remission of 49.5% and 38.9% (versus placebo 29.1% and 21.1%), respectively, and U-ENDURE for the maintenance phase achieved clinical remission rates of 37.3% and 47.6% with the 15mg and 30mg doses compared to 15.1% in the placebo group [57].

## 13. S1p Modulators

S1p modulators act as functional antagonists in lymphocytic receptors, inhibiting lymphocytes dependent on this receptor from leaving secondary lymph nodes for peripheral blood, reducing the circulating number of these cells. There are five variants of the S10 receptor (1, 2, 3, 4, and 5) with different actions, and the action of this therapeutic class will depend on the extent of the blockade [94].

Ozanimod is an oral S1p1 and S1p5 agonist that induces lymphocyte sequestration by peripheral lymph nodes, potently reducing the number of activated lymphocytes circulating to the gastrointestinal tract. The TOUCHSTONE study evaluated the effectiveness of this drug in UC. A total of 19% of the patients in the group using the standard dose had previously been treated with biological drugs. The primary end point was clinical remission, achieved at week 8 by 16% of patients and at the end of 32 weeks (maintenance) by 21% [58].

The ELEVATE UC 12 and ELEVATE UC 52 studies, respectively, evaluated the efficacy of Etrasimod in inducing clinical remission in patients with UC at weeks 12 and 52. At week 12, 25% of Etrasimod users achieved clinical remission, compared to 15% of the placebo group. At week 52, 32% of the patients on etrasimod and 7% of the placebo group achieved clinical remission [59].

## 14. Advances in the Safety of Immunosuppressive Therapies

With the advent of conventional immunosuppressants and later biological therapy and small molecules, concerns have arisen, especially related to infectious events and neoplasms. In an evaluation of clinical studies on the safety of azathioprine and 6-mercaptopurine in the treatment of IBD, it was observed that the therapies are safe if patients are regularly monitored for side effects, especially hepatotoxicity and leukopenia. Although rare, the incidence of non-melanoma skin cancer is increased in users of these immunosuppressants, especially when taken in high doses [96]. Methotrexate has been associated with an increase in respiratory tract infections and is contraindicated in women intending to become pregnant or pregnant women due to its teratogenic effects [97].

Regarding biologicals, the use of anti-TNF agents has revolutionized the treatment of IBD, demonstrating significant benefits in most patients. Although therapeutic risks such as infections, malignancies, and infusion reactions exist, the more than 20 years of use of anti-TNF in IBD and in other immune-mediated diseases have shown that they are safer drugs even than conventional immunosuppression [98]. The most prominent serious adverse events are the risk for tuberculosis disease or latent tuberculosis, the worsening of cardiac functional status in patients previously diagnosed with heart failure and the onset of immune-mediated or demyelinating diseases. The most common adverse events of biologic therapy are injection site reactions and mild infections, such as colds and flu [98,99,100]. The classic combination of infliximab and azathioprine proved to be more effective than the use of the biologic alone, moderately reducing therapeutic safety [79]. Vedolizumab has a selective immunosuppressive action on the gastrointestinal tract and stands out in terms of safety [48,101,102]. Similarly, long-term studies of ustekinumab have shown no increase in the rate of serious infections, as well as no correlation with malignancies [103]. Initial data for risankizumab point to the same safety profile, although long-term studies in the IBD population are needed [104,105].

JAK inhibitors have a lower initial safety profile, although more long-term studies in patients with IBD are needed to corroborate information from other populations, especially those with Rheumatoid Arthritis (RA). A large retrospective cohort involving RA patients using JAK inhibitors showed a significant increase in the risk of lung cancer and lymphoma [95,106,107,108]. These results were not observed in studies involving patients with IBD. Differently, population data of patients with RA using tofacitinib increased the risk of cardiovascular and thromboembolic events and the risk of thrombosis was also evidenced in patients with CD using the same medication, in addition to serious infections and death. These risks, although greater than in the general population, are still low and the therapeutic benefits are greater. It is prudent that patients with this therapeutic indication be carefully selected and monitored [56,107,109,110]. Some regulatory agencies in some countries advise that JAK inhibitors be recommended only in case of failure of a previous biological therapy [111].

Although S1p modulators have shown efficacy in reducing IBD inflammation, there are concerns regarding the long-term safety of these drugs [112]. Studies addressing the safety of S1p modulators in the treatment of IBD are still scarce. Although headache, nasopharyngitis, and elevated liver enzymes were the most common adverse events in the True North studies, viral infections, especially Herpes zoster, were important events [58]. In addition, the medication is contraindicated in patients with heart failure or a recent history of acute myocardial infarction or stroke, patients with cardiac arrhythmias, patients with severe obstructive sleep apnea, or in use of monoamine oxidase inhibitor [113,114,115].

**Figure 5:**
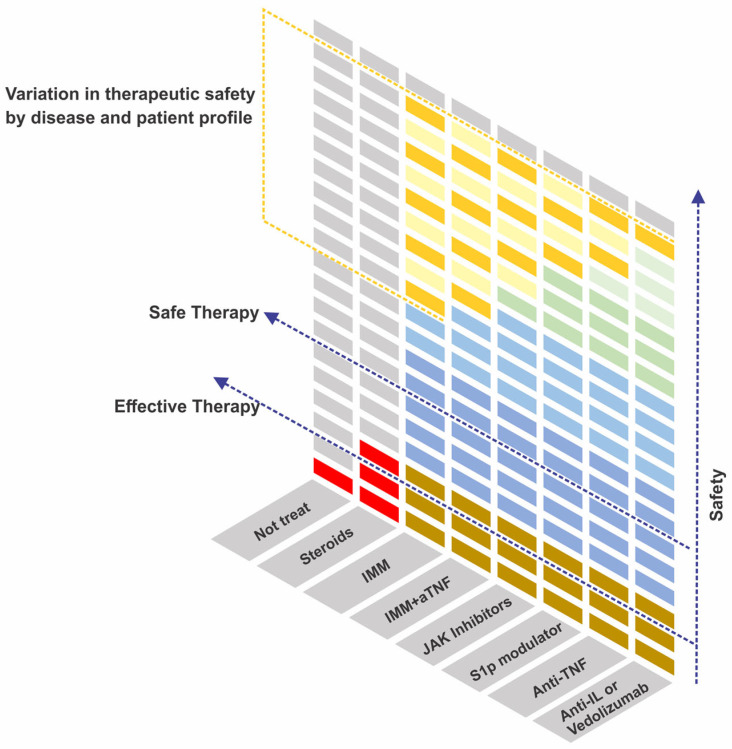
Immunosuppressive therapies for the treatment of inflammatory bowel diseases. The therapies currently proposed for treatment are safe and effective, except for corticosteroids, which in the long term have benefits that are outweighed by the risks. The graph demonstrates a lower to higher safety profile, considering the authors’ opinion. We consider newly approved therapies to be at a disadvantage in this review due to fewer studies addressing long-term safety. Safety between therapies varies by therapeutic class or combination. More importantly, safety varies by subgroup of patient (characteristics such as age and comorbidities) and disease (characteristics such as phenotypes and systemic manifestations) (variation represented by the yellow blocks). The use of safe therapies that are not ideal for the characteristics of the patient’s disease is also a therapeutic risk factor. S1p: Sphingosine 1-Phosphate; JAK: Janus Kinases; IMM: immunomodulators; aTNF: anti-Tumor Necrosis Factor; IL: Interleukins.

Care with the risks associated with the therapy deserves to be adopted, as these are a major concern for patients, but the therapeutic benefits outweigh the risks of the therapy, especially the risks related to the disease [116,117]. As well as the evaluation of therapeutic efficacy, the evaluation of safety is dynamic and changes according to the time of use of each drug and the continuity of observation of patients included in clinical trials (generally incorporated into extension studies) and publications of cohorts of the real world. Therefore, one must consider that the more recent the drugs, the lower the safety data. From this point of view, a recent review of biologics and small molecules used in IBD points to JAK inhibitors and S1p modulators as drugs with a greater safety profile than TNF antagonists, especially considering drugs with greater selectivity [118]. Individualized prescribing can select the best therapy for each individual. Figure 5 outlines that, except for corticosteroids, immunosuppressive therapies go beyond the line of efficacy and therapeutic safety in the treatment of IBD, although safety differs between classes.

## 15. Future Perspectives

The development of new molecules is a reality both for previously approved classes and for new therapeutic classes with different targets. As for TNF antagonists, oral formulations such as AVX-470 and OPRX-106 stand out [119].

While the HIBISCUS and GARDENIA trials found that although Etrolizumab (an anti-integrin monoclonal antibody that binds to the β7 subunit of α4β7 and αEβ7 integrins) was effective in inducing but ineffective in maintaining clinical remission, further analysis identified that microRNAs expressed in patients with UC may indicate patients with a better outcome to this drug [119,120]. Other drugs that act on leukocyte trafficking are under study, such as PN-943, an orally administered α4β7 antagonist, and PF-00547659, a human mAb that binds to a mucosal cell adhesion molecule (MadCAM) [119].

In the class of interleukin blockers, Guselkumab has shown good results in CD treatment in published analyzes of the GALAXI studies [121].

Among small molecules, new therapies with greater selectivity to JAK 1 such as Izencitinib and Peficitinib are under study, in addition to JAK/TYK2 combinations such as Brepocitinib [119].

As for the new mechanisms of action with greater progress in the tests, Phosphodiesterase 4 Inhibitors stand out, where Apremilast has been shown to cause a cyclic adenosine monophosphate breakdown, leading to the activation of the nuclear factor kappa B (responsible for the upregulation of proinflammatory cytokines) [122]. Cobitolimode, a representative Toll-Like Receptor 9 (TLR9) agonist, has demonstrated the ability to induce the expression of IL-1 and type 1 interferon (INF). Currently, Cobitolimode is undergoing phase 3 testing in the CONCLUDE trial [123].

Considering the large number of patients refractory to IBD therapies, the therapeutic combination of biologics and small molecules has been used and disseminated through small real-life studies. In general, the association between different mechanisms of action is chosen [124,125]. The VEGA study evaluated the association of the combination of Golimumab associated with Guselkumab compared to the two drugs in monotherapy with the main objective of evaluating clinical response at week 12, which was achieved by 83%, 61%, and 75% of the groups of combination therapy, Golimumab and Guselkumab, respectively [126]. This therapeutic combination is being evaluated in the DUET studies.

It seems that the therapeutical ceiling in IBD is a reality. This may be due to (i) the inability of novel biologics agents to increase the efficacy of older drugs, (ii) the result of substandard reporting of clinical outcomes in clinical trials designed to adequately test the efficacy, (iii) the result of a wrong strategy when compared to treat-to-target or tight control. Indeed, we need to test new therapeutic strategies such as therapeutic drug monitoring (TDM), intensive treatment based on inflammatory markers, adjusting drug sequencing, use of bispecific antibodies, preventing drug resistance and immune escape, or combination of drugs with different targets, particularly in induction. While the drug combination shows promising efficacy during induction, further investigation is needed to determine if monotherapy during the maintenance phase will be sufficient. Hence, there is a pressing need for innovative strategies that go beyond the development of new drugs, focusing on the identification of effective interventions with similar safety profiles and the establishment of precise biomarkers to closely monitor disease burden in inflammatory bowel disease. These strategies should aim to identify effective targets and strive for achieving disease clearance. Moreover, exploring new and more ambitious targets, such as urgency in ulcerative colitis and quality of life in IBD, is essential to meet the needs felt by the patient.

The limitations of this narrative review of the literature occur due to the authors’ option to describe the launches of therapies for IBD in a linear, temporal way associated with the mechanism of action. In this way, we involved drugs developed before and after the era of clinical trials, making a comparative review of results impossible. Compiling cross-analyses was not intended either.

## 16. Conclusions

The advancement of therapeutic development for IBD has accelerated in the last two decades, but conventional therapies are still widely used, with emphasis on 5-ASA, the first therapeutic class developed, remaining with an important position in the treatment of mild to moderate UC. Despite the different mechanisms of action already released for the treatment of CD and UC, many patients remain unable to achieve remission. The wide phenotypic variation of these diseases may prevent us from identifying individualized actions of each drug for each phenotype since, so far, few trials have been conducted for specific IBD phenotypes. The main outcomes of clinical trials are still focused on clinical remission, while therapy guidelines guide the search for a short-term clinical response and medium- and long-term clinical remission and mucosal healing. Although biological therapies are proven to be safe and effective, the development of small molecules assumes the combination of similar effectiveness with the therapeutic convenience of oral administration. Finally, the development of new therapeutic classes, as well as tests aimed at the combination of biological agents and small molecules, promise to occupy a prominent position in the publications of the next decade. Advances in other therapeutic modalities, such as stem cell transplantation and therapies directed at the microbiota, combined with the introduction of artificial intelligence, can change the scenario of IBD therapy at the end of this century of innovations.

## Figures and Tables

**Table 1:** Drugs recommended in the treatment of IBD [24,25,26,27,28,29,30].

Class	Drug	Disease	Dose		
			Induction	Maintenance	Dose Optimization
Aminosalicylates [26,28,30]	Sulfasalazine	UC	≥3 g/d	≥2 g/d	-
	5-ASA	UC	Distal colitis: rectal 5-ASA ≥ 1 g/d Extensive colitis: rectal 5-ASA ≥ 1 g/d + oral 5-ASA ≥ 2 g/d	≥1 g/d >2 g/d	- >4 g/d
Glucocorticoids [26,27,28,29,31,32]	Prednisolone	CD, UC	0.5–0.75 mg/kg (maximum daily dose 60 mg)	-	-
	Budesonide	CD	9 mg/day 2–3 months	-	-
	Budesonide MMX	UC	9 mg/day 2–3 months	-	-
Immunomodulators [26,27,28,29,30,33]	Azathioprine	CD, UC	1.5–2.5 mg/kg/d		-
	6-Mercaptopurine	CD, UC	1–1.5 mg/kg/d		-
	Cyclosporine	UC	2 mg/kg/d IV	5 mg/kg/d (up to 3 months)	-
	Methotrexate	CD	25 mg/w SC or IM (12w)	15 mg/w SC or IM	-
Anti-TNF	Infliximab [34,35,36,37]	CD, UC	5 mg/kg IV at 0, 2 and 6 w	5 mg/kg IV every 8w 120 mg SC every 2wfrom w6	10 mg/kg IV every 8w (label) or 5 mg/kg every 4w (off-label)
	Adalimumab [38,39,40]	CD, UC	160 mg, then 80 mg after 2w SC	40 mg SC every 2w	80 mg SC every 2w or 40 mg SC weekly
	Certolizumab pegol [41,42]	CD	400 mg SC at weeks 0, 2 and 4	400 mg every 4w	-
	Golimumab [43,44]	UC	200 mg, then 100 mg after 2w SC	50–100 mg SC every 4w	<80 kg using 50 mg: 100 mg every 4w
Anti-Integrin	Natalizumab [45]	CD	300 mg IV every 4w		
	Vedolizumab [46,47,48]	CD, UC	300 mg IV at weeks 0, 2 and 6. *CD: an additional dose at w10 may be indicated*	300 mg IV every 8w 108 mg SC every 2w	300 mg IV every 4w -
Anti-Interleukin	Ustekinumab [49,50]	CD, UC	<55 kg: 260 mg, 55–85 kg: 390 mg, >85 kg: 520 mg IV single dose.	90 mg SC every 12 or 8w	90 mg SC every 4w (off-label)
	Risankizumab [51,52]	CD	600 mg IV at weeks 0, 4 and 8	360 mg SC at w12 and then every 8w	-
	Mirikizumab * [53]	UC	300 mg IV every 4w for 12w	200 mg IV every 4w	
JAK-inhibitors	Tofacitinib [54]	UC	10 mg BID PO 8–12w	5 mg PO BID	In loss of response, consider new induction
	Filgotinib [55]	UC	200 mg PO OD 10w	100–200 mg PO OD	-
	Upadacitinib [56,57]	CD, UC	UC: 45 mg PO OD 8w CD: 45 mg PO OD 12w	15–30 mg PO OD	-
S1p modulators	Ozanimod [58]	UC	Day 1–4: 0.92 mg PO Day 5–7: 0.46 mg PO	0.92 mg PO	-
	Etrasimod * [59]	UC	2 mg PO OD		

Doses referring to the treatment of IBD in adults. * Doses used in phase 3 studies. 5-ASA: 5-aminosalicylates. MMX: multi-matrix system. PO: orally. SC: subcutaneous. IV: intravenous. D: day. W: week.

## Data Availability

Data sharing is not applicable.
